# Surface modification effects on the tensile properties of functionalised graphene oxide epoxy films†

**DOI:** 10.1039/c8ra00252e

**Published:** 2018-03-06

**Authors:** Koji Matsuura, Yuki Umahara, Kazuma Gotoh, Yuko Hoshijima, Hiroyuki Ishida

**Affiliations:** Graduate School of Natural Science and Technology, Okayama University 3-1-1 Tsushima-Naka Kita-Ku Okayama 700-8530 Japan kmatsuura@bme.ous.ac.jp; Department of Biomedical Engineering, Faculty of Engineering, Okayama University of Science 1-1 Ridai-Cho Kita-Ku Okayama 700-0005 Japan

## Abstract

Graphene oxide (GO) is a candidate for nanofillers to improve the mechanical and thermal stability of nanocomposites. In order to determine the molecular interaction to improve the mechanical properties of GO–epoxy resin composites, we investigated the relationship between GO oxidation properties and the tensile strength of the epoxy resin. With respect to GO preparation, graphite was oxidised by the Brodie or Hummers method, and the oxidised GO was reduced or chloride substituted. The X-ray photoelectron spectroscopy (XPS) spectral patterns indicate that a shorter Brodie oxidation method GO (B-GO) is associated with a higher proportion of hydroxyl groups. The oxidised GO materials, with the exception of the sample produced by the 54 h Brodie oxidation method, improved the tensile strength of the composites while the epoxy resin with reduced or chlorinated GO did not increase the tensile strength of the film. Based on XPS and elemental analyses, the improvement in the tensile strength is due to the presence of O atom based functional groups, such as hydroxyl groups, on the GO surface. The interaction between the epoxy resin and O atom based functional groups on the GO contributes to improving the tensile strength of the composites.

## Abstract 2

In order to determine the molecular interaction to improve the mechanical properties of graphene oxide (GO)–epoxy resin composites, we investigated the relationship between GO oxidation properties and the tensile strength of the epoxy resin.

## Graphical abstract

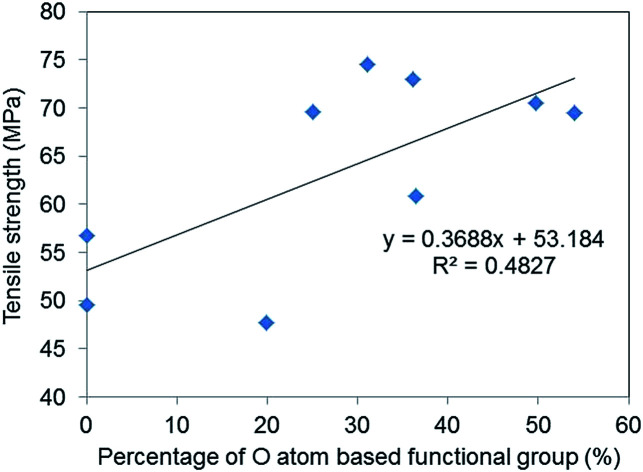

## Introduction

Epoxy resin is a thermosetting polymer that is used as a matrix that is hardened by a crosslinking reaction between the epoxy groups of the monomer and amine groups of the hardener. It is currently used for coating materials in electrical circuits and devices due to its high electrical insulation properties and is used as a bonding agent to prevent the occurrence of cracking in the concrete used for buildings.^1^ In order to improve the strength of epoxy resins, nanocomposites were developed.^2–21^ However, in the case of inorganic nanofillers, the difficulties in the mono-dispersion of the fillers in the hydrophobic resin results in brittleness due to the low dispersibility of the filler. Therefore, relatively hydrophobic nanocarbon based fillers including carbon nanotubes, fullerene, graphene and graphene oxide (GO) are added to epoxy resins.^2^ Specifically, GO is prepared by the oxidation of graphite powder by nitric acid or sulfonic acid, and the synthesis method is suitable for scaling-up as opposed to the oxidation of carbon nanotubes and other carbon nanomaterials. Furthermore, GO includes several chemical functionalisation processes to their surface and edge.^5–21^ Therefore, mechanical and chemical properties of the polymer composites are tuned based on the amount and contents of GO functional groups.

In order to enhance the crosslink network between the amine functionalised nanofiller and epoxy monomer for GO–epoxy composites, the aliphatic and aromatic amine groups were functionalised to the GO edge, and the tensile strength and glass transition temperature of the composites that contained the functionalised GO improved.^5–19^ When GO was grafted with epoxy chains, the improvement in tensile strength were also observed due to changes in the curing behaviour.^20,21^ However, in order to extend this technology for large scale application, the scalability of GO functionalisation processes acts as an obstacle for amine functionalisation and GO grafting methods. We suggest that molecular scale interaction between GO and epoxy resin contributes to designing GO-based composites for large scale production. The use of oxidised or reduced GO as a nanofiller allows the discussion of mechanical properties of GO-based composites based on the molecular interaction between GO and epoxy function groups.

In the present study, we discuss the relationship between degree of GO oxidation and tensile strength of the GO–epoxy resin composites with the aim of obtaining a critical interaction between GO filler and epoxy resin to improve the mechanical strength of GO–epoxy resin composites. In order to investigate the influence of GO functional groups on the tensile strengths of the composites, GO was prepared by employing the Brodie method or Hummers method and the dispersion of GO samples with different degrees of oxidation including reduction treatment and chloride substitution, preparation of GO thermosetting epoxy resin composite films, and tensile tests of the films.

## Materials and methods

### Materials used in the study

Graphite powder, potassium chlorate, sulfuric acid, KMnO_4_, hydrazine monohydrate and thionyl chloride were purchased from Wako Chemical Co. Ltd. (Osaka, Japan). 94% Fuming nitric acid, hydrogen peroxide and *N*,*N*-dimethylformamide (DMF) were obtained from Nakarai tesque Co. Ltd. (Kyoto, Japan).

### GO synthesis and functionalisation methods

#### Brodie method

Specifically, 0.5 g of graphite powder was mixed with 10 mL of white fuming nitric acid and 4 g of potassium chlorate and oxidised for 0.5 h, 3 h or 54 h.^22^ The black suspension was diluted with 250 mL of deionised water and centrifuged at 8000 rpm for 20 min. Following the removal of supernatants, deionised water was added and centrifuged until the suspension obtained a pH of 7. The suspension was filtrated and dried (60 °C, 18 h). We obtained 0.5 h oxidised GO (0.5hB-GO), 3 h oxidised GO (3hB-GO) and 54 h oxidised GO (54hB-GO) with different degrees of oxidation.

#### Hummers method

In this method, 0.5 g of graphite powder was mixed with 15 mL of sulfuric acid and 0.5 g (1 equivalent), 1.5 g or 2.5 g of KMnO_4_ and oxidised for 2 h at 35 °C.^23^ In order to quench the oxidation of graphite, distilled water (15 mL) and hydrogen peroxide (1.5 g) were added to the suspension and reacted for 30 min. The suspension was centrifuged at 8000 rpm for 20 min. Following the removal of supernatants, deionised water was added and centrifuged until the pH of the GO suspension corresponded to 7. The resultant powder was filtrated and dried up at 100 °C for 18 h. When the ratios of graphite/oxidant were 1, 1/3 or 1/5, the respective final products were defined as 1H-GO, 3H-GO or 5H-GO, respectively.

#### Thermal reduction

In this method, 0.5hB-GO was heated in a tube furnace under a N_2_ atmosphere (400 °C, 30 min), and the resultant material is thermally reduced GO (T-rGO).

#### Hydrazine reduction

Specifically, 0.5hB-GO powder and filtration paper soaked with 2 mL of hydrazine monohydrate were sealed in a glass flask by using a paraffin film. We obtained hydrazine reduced GO (Hyd-rGO) by exposing GO to hydrazine vapour at 90 °C for 6 h.^24^

#### Chloride substitution

In this method, 2 mL of thionyl chloride was added to the 0.5hB-GO or 54hB-GO suspension. Additionally, DMF that was used as a catalyst was added in a dropwise manner to the suspension and sonicated for 30 min. The mixture was refluxed under N_2_ atmosphere (60 °C, 42 h). The refluxed mixture was cooled to room temperature, filtered at 60 °C, and dried at room temperature. The final product is termed as chloride substituted GO (0.5hCl-GO or 54hCl-GO) corresponding to the oxidation time of B-GO. Information about the GO sample is presented along with the acronyms in Table 1.

Summary of GO samples that were used in this study. The numbers that are underlined are related to the acronyms

**Table:** 

	Treatment time (h)	Ratios of graphite/oxidant	Acronyms
Brodie method (B-GO)	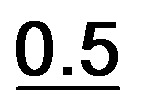	8	0.5hB-GO
	**3̲**	8	3hB-GO
	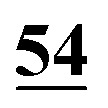	8	54hB-GO
Hummers method (H-GO)	2	1 : **1̲**	1H-GO
	2	1 : **3̲**	3H-GO
	2	1 : **5̲**	5H-GO

**Table:** 

	Reduction method	Acronyms
Reduced GO (rGO)	Thermal reduction	T-rGO
	Hydrazine reduction	Hyd-rGO

**Table:** 

	Initial GO sample	Acronyms
Cl substituted GO	0.5hCl-GO	0.5hCl-GO
	54hCl-GO	54hCl-GO

### GO characterisation (XRD, XPS and elemental analyses)

Particle size of GO samples was analysed by using an optical microscope (Keyence, VX-100, Osaka, Japan) and the average length was approximately 20 (μm) for the optical microscopically observed samples. We did not observe any difference between the sizes of GO in different oxidation processes using an optical microscope, as depicted in Fig. S1A and B in ESI.† We recorded X-ray diffraction (XRD) spectra of 0.5hB-GO, 3hB-GO, 54hB-GO, 1H-GO, 3H-GO and 5H-GO by using a powder XRD method with a diffractometer (MiniFlex II, Rigaku, Tokyo, Japan) and CuKα radiation (*λ* = 1.54184 Å).^25^Eqn (1) was used to calculate the interlayer distance of the graphene sheet of GO.

In 0.5hB-GO, 3hB-GO, 54hB-GO, 1H-GO, 3H-GO, 5H-GO, T-rGO, Hyd-rGO, 0.5hCl-GO and 54hCl-GO of the GO samples, the GO functional groups and the percentages were analysed. The X-ray photoelectron spectroscopy (XPS) spectra were recorded by using XPS (JPS-9200, JEOL, Tokyo, Japan) and curve-fitted by a gauss function by using IGOR Pro (WaveMetrics, Inc., Portland, OR). Hydroxyl group contents were calculated by comparing the peak areas of 286 eV and 282 eV in the XPS spectra.

The elemental analysis of GO samples was performed at the Okayama University Advanced Science Research Centre, Department of Instrumental Analysis. We estimated the percentages of O atoms based on carbon, hydrogen and nitrogen (C, H and N) atomic component ratios and compared the percentage of O atoms in the GOs. The contents of the hydroxyl group of the samples were calculated based on the calculations of the O atom percentage and hydroxyl group ratio from the analyses of XPS spectra ([hydroxyl group ratio by XPS]) as shown in eqn (1).

1 [contents of hydroxyl group] = [percentage of O atom] × [hydroxyl group ratio by XPS]/100

### Preparation of epoxy composite films and tensile tests

Specifically, 500 μl of DMF or GO suspension was added to 3 g of epoxy resin monomer (JER® grade 828, Mitsubishi Chemical, Tokyo, Japan), mixed for 1.5 min by using a deaerator (CR-100, Thynky Co. Ltd., Tokyo, Japan). Additionally, 2 g of epoxy resin hardener (JER cure® grade ST11; aliphatic amine-based hardener, Mitsubishi Chemical, Tokyo, Japan) was also added to the GO–epoxy monomer suspension, mixed by using deaerator and degassed for 20 min by using a vacuum pump. In order to prepare cured epoxy sheet, the resin was added on a silicone sheet and degassed for 10 min. The degassed resin was sandwiched by using two silicone sheets, and a weight of 500 g was placed on the silicone sheet containing the resin for 10 min. The resin was incubated in a heater at 80 °C for 5 h, and it was completely cured at 30 °C for 18 h. Transmission images of a 0.05 wt% GO composite using an optical microscope depict very little aggregate of GO inside the film, as depicted in Fig. S1C and D of ESI.† Following the curing, test pieces of the GO–epoxy film with a thickness approximately in the range of 0.08–0.15 mm were prepared. Tensile strength, elastic modulus and strain of the test pieces were investigated by using a tensile test machine (SVZ-50NA-2, Imada Seisakusyo Co. Ltd., Tokyo, Japan). Tensile properties (tensile strength, elastic modulus and fracture strain) of the GO films were compared with those of the neat film prepared on the same day. Even though the curing processes were observed to be similar between these conditions, the tensile strengths of neat epoxy samples would be dependent on the room temperature that differs in seasons (summer or winter) during the curing process.

## Results and discussion

### XRD

**Fig. 1:**
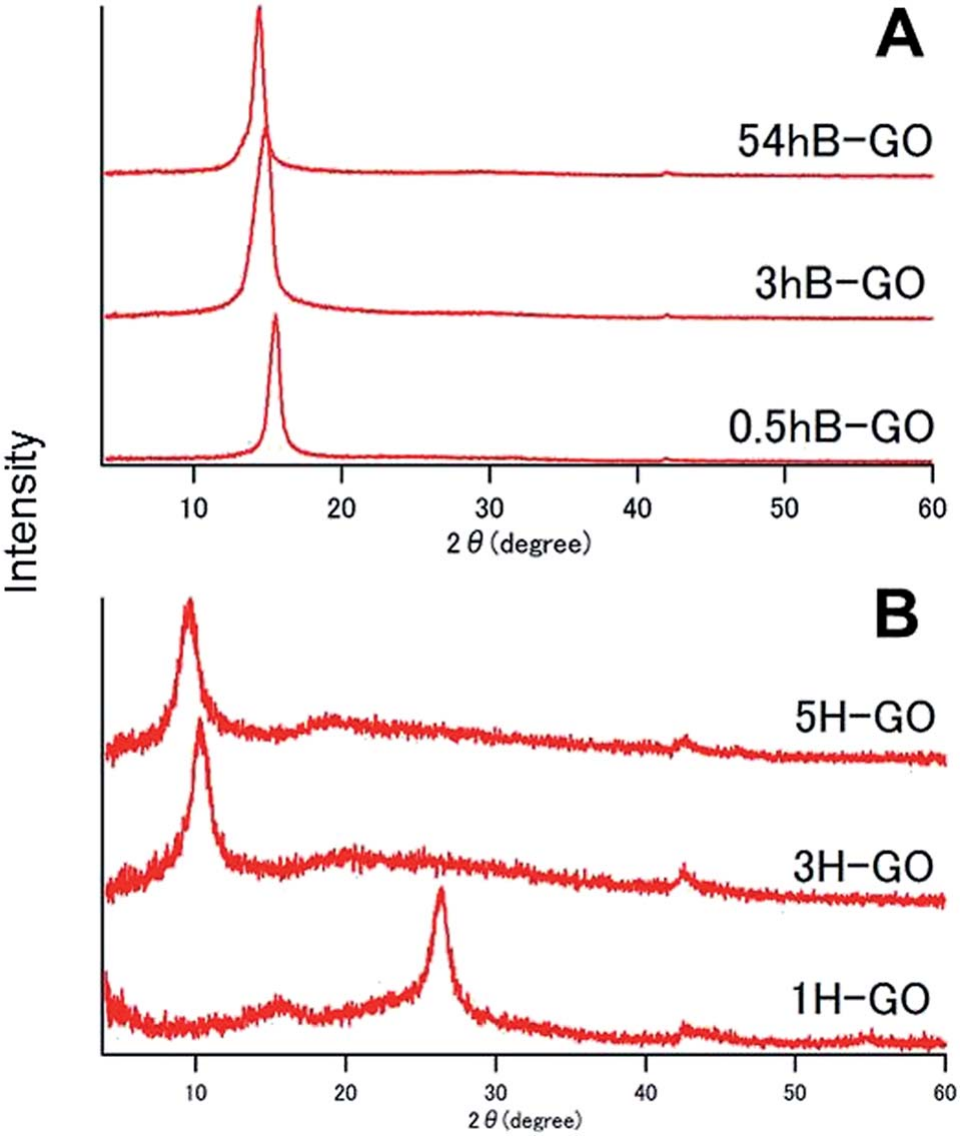
XRD patterns of (A) B-GO and (B) H-GO.

Fig. 1 shows XRD spectra of GOs as prepared by Brodie and Hummers method. In the spectra for 0.5hB-GO, 3hB-GO and 54hB-GO, peaks at 2*θ* = 15° were observed without a peak at 2*θ* = 26° from graphite, suggesting graphite oxidation. Peaks corresponding to 2*θ* = 10° were also observed in XRD spectra of 3H-GO and 5H-GO, while peaks corresponding to 2*θ* = 26° and 15° were observed in the spectrum of 1H-GO, thereby suggesting the occurrence of stronger oxidation to increase the distance between graphene sheets with unoxidised graphite species.

### XPS

**Fig. 2:**
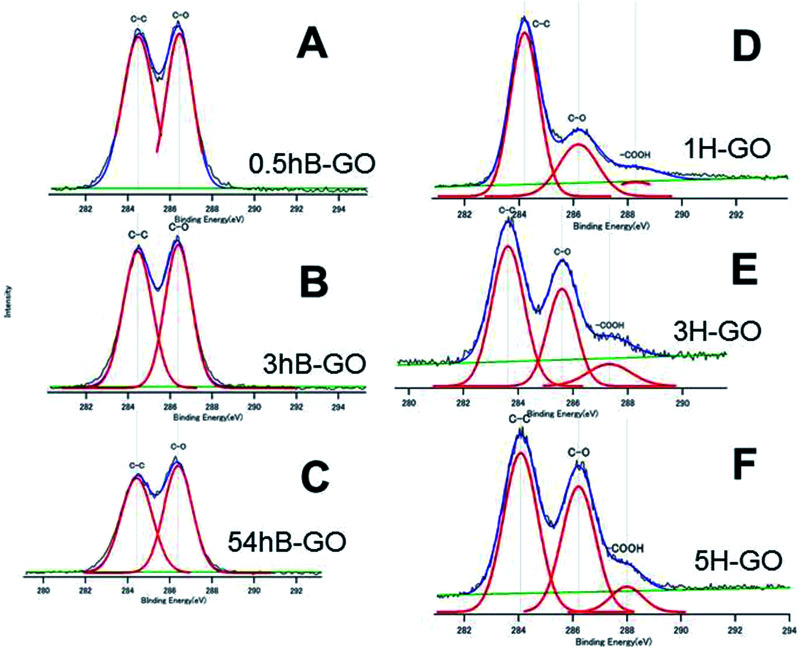
The XPS C1s spectra of (A) 0.5hB-GO, (B) 3hB-GO, (C) 54hB-GO, (D) 1H-GO, (E) 3H-CO and (F) 5H-GO. Deconvoluted curves indicate the existence of the respective chemical bonds.

Fig. 2 shows C1s spectra of GOs oxidised by Brodie and Hummer's method. Peaks at approximately 284 eV, 286 eV and 288 eV are attributed to C–C, C–O (hydroxyl or epoxy group) and carboxyl groups, respectively. Additionally, O and C atomic ratios (O/C ratio) calculated from peak area and those for 0.5hB-GO, 3hB-GO and 54hB-GO were 1.77, 1.95 and 2.06, respectively, and this corresponds to the progression of graphite oxidation. The O/C ratios of 1H-GO, 3H-GO and 5H-GO were 1.09, 2.23 and 2.57, respectively. Increases in the reaction time and oxidant concentration increase the O/C ratio of the GO.

**Fig. 3:**
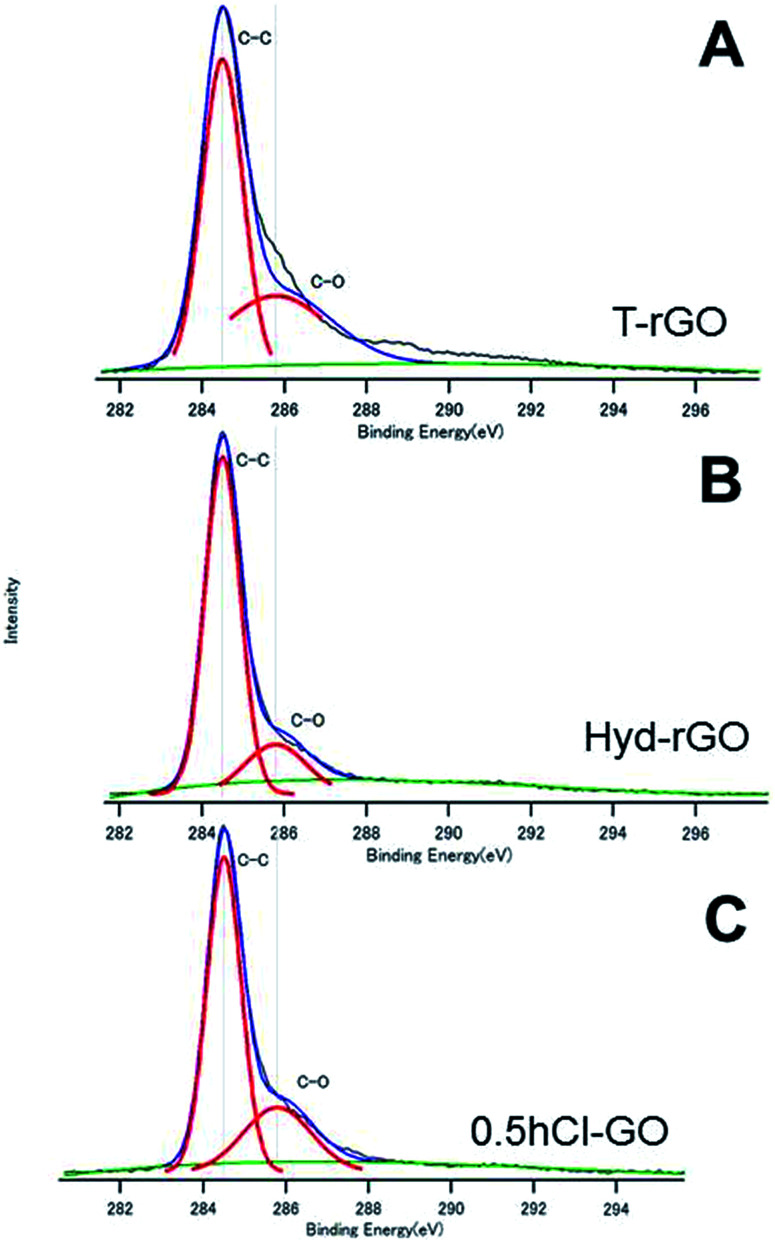
The XPS spectra of (A) T-rGO, (B) Hyd-rGO and (C) 0.5hCl-GO. The deconvoluted curves indicate the existence of respective chemical bonds.

Fig. 3A and B show the C1s spectra of T-rGO and Hyd-rGO prepared by the reduction of 0.5hB-GO, respectively. Both O/C ratios of T-rGO and Hyd-rGO corresponded to 0.33, and this suggests the reduction of 0.5hB-GO (O/C ratio 1.77) by both thermal or hydrazine methods. Fig. 3C shows the C1s spectra of chloride substituted Cl-GO. Chloride substitution of the sample was confirmed by observations of Cl–C peak around 200 eV in the Cl2p3/2 spectra. Reduction of O/C ratio = 0.56 was due to a decrease in the O atom in the hydroxyl group.

**Fig. 4:**
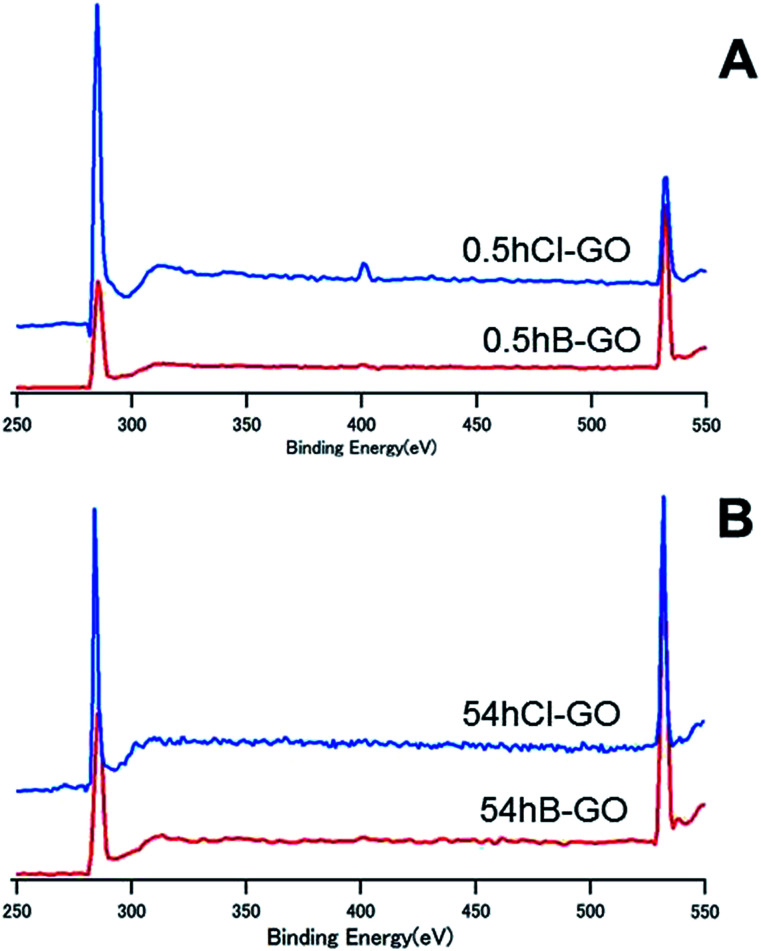
The XPS spectra of (A) 54hCl-GO and (B) 0.5hCl-GO from 250 to 550 eV.

Fig. 4 shows the XPS spectra of 0.5hB-GO, 0.5hCl-GO, 54hB-GO and 54hCl-GO from 250 eV to 550 eV. Peaks at approximately 280 eV and 530 eV are attributed to C and O atoms, respectively. Chloride substitution of the 0.5hB-GO decreases the O/C ratio from 1.77 to 0.56. When all hydroxyl groups were substituted to chloride groups, the calculated percentage of hydroxyl group in 0.5hB-GO was approximately 68% of the oxygen-based functional groups. The O/C ratio was 1.09 with respect to chloride substitution of 54hB-GO, and this suggests that the hydroxyl group percentage among oxygen-based functional groups was calculated as approximately 47% in 54hB-GO. The percentage of hydroxyl group in 0.5hB-GO exceeded that in 54hB-GO.

### Elemental analyses

Table 2 shows the CHN elemental analysis (wt%) and estimated percentages of O atoms. It is not possible to estimate the percentage of O atoms of 0.5hCl-GO and 54hCl-GO from the percentages of CHN atoms since they contain Cl atoms. The N atom of Hyd-rGO is derived from hydrazine monohydrate. Additionally, N and H atoms of 0.5hCl-GO and 54hCl-GO correspond to residual DMF, which is used as a catalyst with thionyl chloride during the chloride substitution. Trace amounts of N atoms in the 5H-GO sample are potentially due to impurities.

**Table:** Results of the elemental analyses for C, H and N atoms of the prepared GO samples and the estimated percentage of O atoms based on the elemental analysis

		C%	H%	N%	O%
B-GO	0.5hB-GO	68.11	0.80	—	31.09
	3hB-GO	62.28	1.41	—	36.16
	54hB-GO	62.10	1.42	—	36.48
H-GO	1H-GO	74.03	0.87	—	25.1
	3H-GO	43.64	2.33	—	54.03
	5H-GO	45.23	2.85	2.22	49.7
rGO	Hyd-rGO	70.00	Less than 0.1	10.06	19.94
Cl substituted GO	0.5hCl-GO	53.24	5.24	8.91	Cannot calculated
	54hCl-GO	43.17	2.68	3.24	Cannot calculated

The O atom percentages for 0.5hB-GO and 54hB-GO were 31.09% and 36.48%, and the O atom percentages for hydroxyl group (wt%) of 0.5hB-GO and 54hB-GO were 21.3% and 17.1%, respectively. The results suggest that oxidised GO contains relatively higher contents of hydroxyl groups in the early stage of Brodie method, and the percentage of the epoxy group increases with increases in oxidation duration. The O atom percentage of 1H-GO was 25.1%, and this is lower than those of other oxidised GO samples.

A decrease in the H atom contents of 0.5hB-GO and 1H-GO when compared to those of other oxidised samples was due to the withdrawal of a few functional groups at the surface by vacuum drying for elemental analysis. When all the O atoms of the GO with an oxidation duration exceeding 2 h correspond to the hydroxyl group, the ratio of the hydroxyl group to the carboxyl group was calculated to be 3 : 1 from the peak area of XPS spectra. The O atom percentage of Hyd-rGO was 19.94%, and the H atom content was less than 0.1%, thereby suggesting the elimination of hydroxyl group in Hyd-rGO from 0.5hB-GO.

### Tensile tests of GO–epoxy composite films

**Fig. 5:**
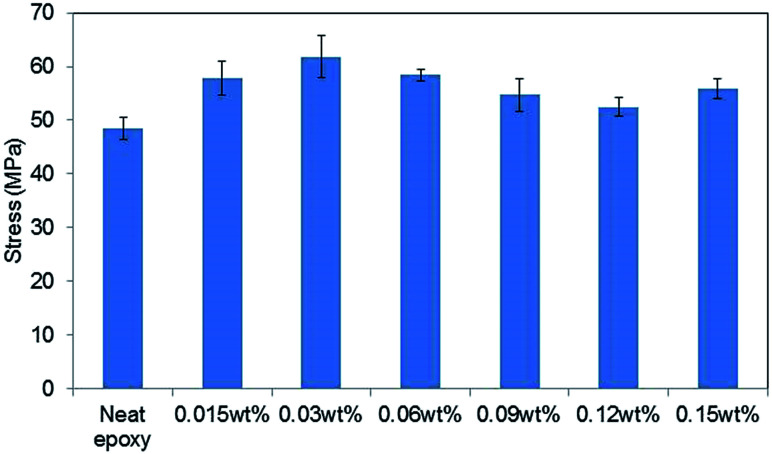
Concentration effects on the tensile stress of GO epoxy composite film containing 0.5hB-GO. Error bars denote the standard deviation.

Fig. 5 shows the tensile strengths of GO–epoxy composites containing 0.015–0.15 wt% of 0.5hB-GO. The tensile strength of neat epoxy resin was below 50 MPa, and tensile strength of GO–epoxy resin composites containing 0.015–0.15 wt% approximately improved by 20% from that of neat epoxy resin.

When the GO concentration in cured epoxy resin exceeded 0.3 wt%, the tensile strengths of the GO–epoxy resin composites were same as those of neat epoxy resin or below those of neat epoxy resin. Suitable GO concentration for the tensile strength improvement corresponded to 0.015–0.06 wt%. The optimised concentration for maximum strength of the test piece was similar to those obtained in extant studies (0.1%).^9,20^ According to previous studies, as the dispersibility of the GO-based filler in a resin improved, the tensile strength of the composite increased.^26^ Because we could not observe any significant difference in the sizes of GO-based fillers based on the oxidising conditions, the concentration dependence that is depicted in the filler could be related with the filler dispersibility of the resin.

**Fig. 6:**
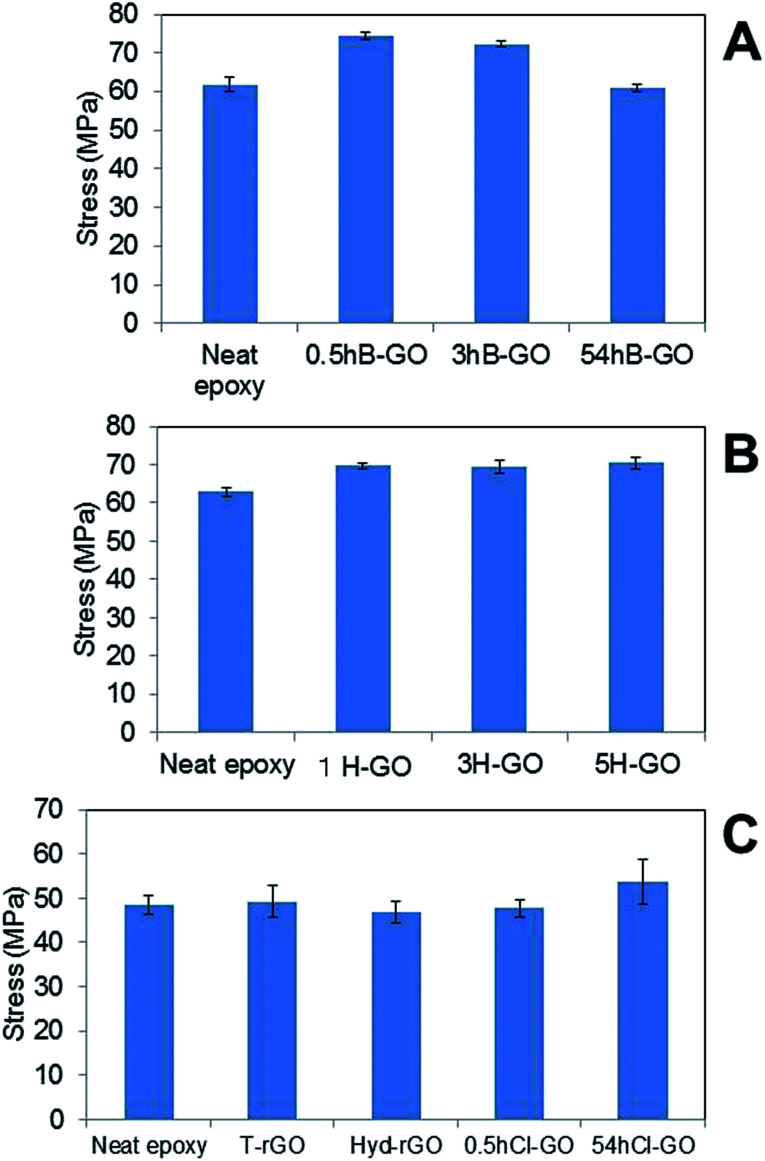
Comparison of tensile stress of GO epoxy composite film between neat epoxy film containing (A) B-GO, (B) H-GO and (C) rGO and chloride substitute GO. Error bars denote the standard deviation.

Fig. 6A and B show the average tensile strengths of GO–epoxy resin composites containing 0.6 wt% B-GO and H-GO, respectively. We confirmed that the addition of 0.5hB-GO and 3hB-GO improves tensile strengths of the GO–epoxy resin composites. However, tensile strengths of GO–epoxy resin composites with 0.06 wt% 54hB-GO were the same as those of neat epoxy resin. The results suggest that 0.5hB-GO and 3hB-GO contain a few components to improve tensile strength and that amount of the components in 54hB-GO must be less than those of 0.5hB-GO and 3hB-GO. The polar interaction between 54hB-GO and epoxy resin would become weak owing to the decrease in the hydroxyl group with respect to 54hB-GO because the XPS spectra and elemental analyses suggest that the contents of the hydroxyl group in 54hB-GO decrease as the oxidation treatment time increases during the Brodie method. The results indicate a tensile strength improvement in GO–epoxy resin composites with respect to the Hummers method (1H-GO, 3H-GO, and 5H-GO). Although the unoxidised graphite domain remained in 1H-GO, the mixture of 1H-GO with the epoxy resin improved the tensile strength such that it was same as that of 3H-GO and 5H-GO–epoxy composites. All H-GO samples used in the study contained components to improve the tensile strength.

Fig. 6C shows the average tensile strengths of GO–epoxy resin composites containing 0.06wt% T-rGO, Hyd-rGO, 0.5hCl-GO, and 54hCl-GO. Reduction of 0.5hB-GO induces a decrease in the tensile strength to the value of neat epoxy resin. Functional groups of GO surface influence tensile strength of the GO–epoxy composites. We consider the tensile strength of epoxy resin composite containing 54hCl-GO as equivalent to that of neat epoxy resin (*t*-test: *P* = 0.03) because it is assumed that *P* < 0.05/5 = 0.01 corresponds to a significant difference with respect to the comparison of the five groups (neat *versus* other samples) as shown in Fig. 6C. The addition of chloride substituted GO to epoxy resin does not improve tensile strength, and functional groups that improve the tensile strength of the GO–epoxy composites correspond to the withdrawn hydroxyl group by chloride substitution.

Table 3 shows the tensile strengths, fracture strains and elastic moduli of neat epoxy resin and GO–epoxy resin composites. Fracture strains of the samples ranged from 4% to 7%. Elastic moduli of the composites were approximately in the range of 2–3 GPa, and this is similar to that of conventional neat epoxy resin. In the composites containing 54hB-GO and 54hCl-GO, the elastic moduli increased by 17% and 22%. The reason for this increase is the enhancement in the cross-linking amine group of hardener with the epoxy group of the GO sample.

**Table:** Results of tensile tests of GO–epoxy films. Values in the tables denote the division of parameters of GO–epoxy films by those of the neat epoxy film (average ± standard deviation (*N* = 3)). The average and standard deviations are compared with those of the neat samples

		Tensile Strength	Fracture Strain	Elastic Modulus
B-GO	0.5hB-GO	1.21 ± 0.50	1.09 ± 0.38	0.96 ± 5.96
	3hB-GO	1.18 ± 0.15	1.21 ± 1.30	1.00 ± 3.45
	54hB-GO	0.98 ± 0.46	0.93 ± 0.094	1.17 ± 0.70
H-GO	1H-GO	1.11 ± 1.00	0.82 ± 0.31	0.82 ± 18.9
	3H-GO	1.11 ± 1.86	1.10 ± 0.21	0.93 ± 12.4
	5H-GO	1.12 ± 1.75	0.81 ± 0.36	0.97 ± 25.1
rGO	T-rGO	1.07 ± 1.34	0.59 ± 0.19	1.08 ± 0.42
	Hyd-rGO	0.99 ± 0.48	0.88 ± 1.02	1.02 ± 0.92
Cl substituted GO	0.5hCl-GO	1.02 ± 0.61	1.01 ± 2.12	0.94 ± 1.02
	54hCl-GO	1.17 ± 1.83	0.72 ± 0.56	1.22 ± 0.67

**Fig. 7:**
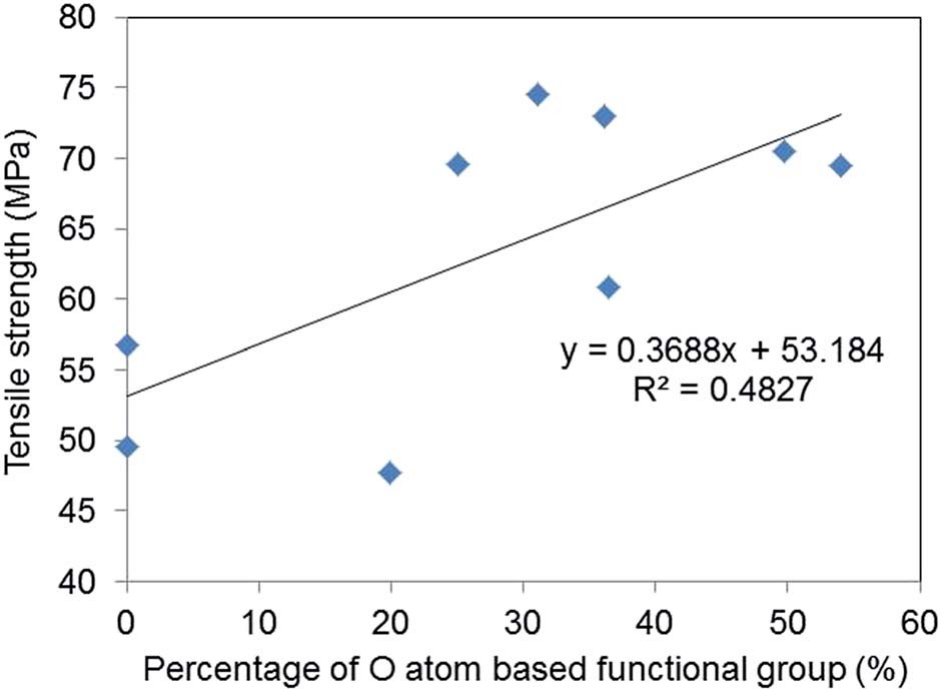
Correlation between the percentages of O atom-based functional group of GOs and the average tensile strengths of the GO epoxy composite films.

Fig. 7 shows the relationship between O atom contents and tensile strength and fracture strain and suggests that a weak correlation exists between oxygen-based functional group content and the tensile strength of GO–epoxy resin composites (*R*^2^ = 0.48). It is reported that enhanced interaction/adhesion was observed in the GO composites owing to the oxygen functional groups and the winkled and rough surface of the filler by comparing the tensile properties of graphene and GO composites, which are consistent with this week correlation, as depicted in Fig. 7.^27^ The increasing surface area of GO also contributed to the improvement in the tensile strengths of the composites because of the increasing van der Walls interaction.^28,29^ Furthermore, the encapsulation of graphene or rGO with surfactants can improve the tensile strength of GO–epoxy composites owing to the dispersibility and non-covalent interaction between the filler and resin.^30,31^ Additionally, the GO-based filler sizes that are prepared by milling and/or functionalisation processes influenced the strengths and toughness of the GO–epoxy composites.^26,32,33^ In this study, we consider that the size effects would not considerably impact the tensile strengths of GO-based composites based on GO sizes and dispersibility in the epoxy resin because we used the same graphite powders as the initial material for the GO preparation. The enhancement in the tensile strength of our composites could be attributed to both covalent and non-covalent interactions of the oxygen-based functional groups.

We also discuss the manner in which the hydroxyl group improves the tensile strength of GO–epoxy resin composites due to hydrogen bonds between GO and epoxy resin of amino group of the curing agent or the hydroxyl group of the monomer. Bonding energy between hydroxyl group of epoxy resin and carbon fibre surface was 13.8 kcal mol^−1^ as indicated by the density functional theory calculation.^34^ The energy was considerably similar to the bonding energy between epoxy resin and oxidised aluminium surface, and this suggests that the interaction between carbon fibre and epoxy resin resembles those of oxidised aluminium/epoxy resin by hydrogen bonds. The tensile strength improvement in GO–epoxy resin composites is due to hydrogen bonds between hydroxyl group at GO surface and polar groups (hydroxyl and amine groups) in epoxy resin. Furthermore, the bonding energy of hydrogen bonds between carboxyl group of filler and hydroxyl group of epoxy resin increased to 19.1 kcal mol^−1^ and exceeded that of the energy containing hydroxyl group.^34^ Therefore, we hypothesise that H-GO with hydroxyl group and carboxyl group are preferable with respect to improving the tensile strength of epoxy resin.

## Conclusions

In this study, we compared the tensile strengths of GO–epoxy films containing oxidised GO and GO after chemical modification. The results indicated that the tensile strengths of GO–epoxy resin composites containing T-rGO, Hyd-rGO, 0.5hCl-GO and 54hCl-GO were similar to those of epoxy resin without GO. The tensile strength improvement by GO addition to epoxy resin was due to hydroxyl groups at the GO surface. The findings suggest that oxidised GO containing hydroxyl group is appropriate for improved GO–epoxy resin composites. Additionally, H-GO contains both hydroxyl group and carboxyl group, and these polar functional groups are expected to contribute to tensile strength improvement.

## Conflicts of interest

There are no conflicts to declare.

## Supplementary Material

RA-008-C8RA00252E-s001
