# Supplementary material for: Surface modification effects on the tensile properties of functionalised graphene oxide epoxy films
Source: RSC Adv. 2018 Mar 6;8(18):9677–84. doi: 10.1039/c8ra00252e (PMC9078660; doi:10.1039/c8ra00252e)
Supplement: RA-008-C8RA00252E-s001 [file RA-008-C8RA00252E-s001.pdf]

Electronic Supplementary Material (ESI) for RSC Advances.
This journal is © The Royal Society of Chemistry 2018

Electronic Supplementary Information (ESI)

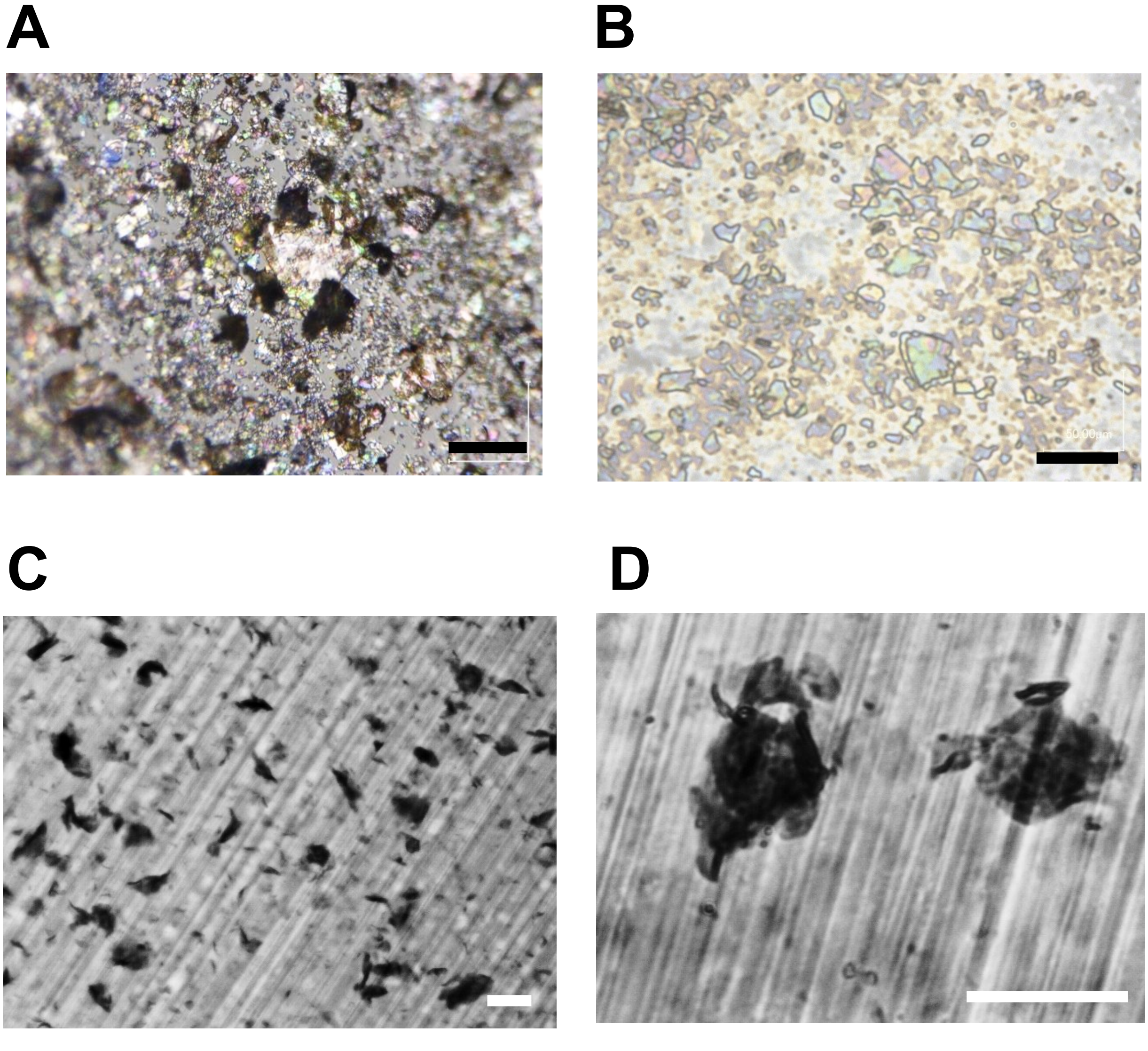

SFig. 1 Optical microscopic images of (A) 3hB-GO; (B) GO prepared according to the Hummers method with a pre-oxidation process using P_2O_5 and (C) GO fillers of (B) dispersed in the epoxy film. (D) corresponds to the magnified image of (C). The GO concentration in the epoxy composite (as shown in (C) and (D)) is 0.05wt%. The bars in the bottom right corners correspond to 50 μm.
